# Respiratory Commensal Bacteria *Corynebacterium pseudodiphtheriticum* Improves Resistance of Infant Mice to Respiratory Syncytial Virus and *Streptococcus pneumoniae* Superinfection

**DOI:** 10.3389/fmicb.2017.01613

**Published:** 2017-08-23

**Authors:** Paulraj Kanmani, Patricia Clua, Maria G. Vizoso-Pinto, Cecilia Rodriguez, Susana Alvarez, Vyacheslav Melnikov, Hideki Takahashi, Haruki Kitazawa, Julio Villena

**Affiliations:** ^1^Food and Feed Immunology Group, Laboratory of Animal Products Chemistry, Graduate School of Agricultural Science, Tohoku University Sendai, Japan; ^2^Livestock Immunology Unit, International Education and Research Center for Food and Agricultural Immunology, Graduate School of Agricultural Science, Tohoku University Sendai, Japan; ^3^Immunobiotics Research Group Tucuman, Argentina; ^4^Laboratory of Immunobiotechnology, Reference Centre for Lactobacilli (CERELA-CONICET) Tucuman, Argentina; ^5^Faculty of Medicine, INSIBIO (UNT-CONICET), National University of Tucuman Tucuman, Argentina; ^6^Laboratory of Genetics, Reference Centre for Lactobacilli (CERELA-CONICET) Tucuman, Argentina; ^7^Gabrichevsky Institute of Epidemiology and Microbiology Moscow, Russia; ^8^Central Research Institute of Epidemiology Moscow, Russia; ^9^Laboratory of Plant Pathology, Graduate School of Agricultural Science, Tohoku University Sendai, Japan; ^10^Plant Immunology Unit, International Education and Research Center for Food and Agricultural Immunology, Graduate School of Agricultural Science, Tohoku University Sendai, Japan

**Keywords:** *Corynebacterium pseudodiphtheriticum*, TLR3, Respiratory Synsytial Virus, *Streptococcus pneumoniae*, respiratory immunity, nasal probiotic

## Abstract

*Corynebacterium pseudodiphtheriticum* is a Gram-positive bacterium found as a member of the normal microbiota of the upper respiratory tract. It was suggested that *C. pseudodiphtheriticum* may be potentially used as a next-generation probiotic for nasal application, although no deep studies were performed in this regard. We hypothesized that human isolate *C. pseudodiphtheriticum* strain 090104 is able to modulate the respiratory innate immune response and beneficially influence the resistance to viral and bacterial infections. Therefore, in the present study we investigated how the exposure of infant mice to nasal priming with viable or non-viable *C. pseudodiphtheriticum* 090104 influences the respiratory innate immune response triggered by Toll-like receptor (TLR)-3 activation, the susceptibility to primary Respiratory Synsytial Virus (RSV) infection, and the resistance to secondary *Streptococcus pneumoniae* pneumonia. We demonstrated that the nasal priming with viable *C. pseudodiphtheriticum* 090104 differentially modulated TLR3-mediated innate antiviral immune response in the respiratory tract of infant mice, improving their resistance to primary RSV infection, and secondary pneumococcal pneumonia. In association with the protection against RSV-pneumococcal superinfection, we found that viable *C. pseudodiphtheriticum* improved lung CD3^+^CD4^+^IFN-γ^+^, and CD3^+^CD4^+^IL-10^+^ T cells as well as CD11c^+^SiglecF^+^IFN-β^+^ alveolar macrophages. Of interest, non-viable bacteria did not have the same protective effect, suggesting that *C. pseudodiphtheriticum* colonization is needed for achieving its protective effect. In conclusion, we present evidence that nasal application of viable *C. pseudodiphtheriticum* could be thought as an alternative to boost defenses against RSV and secondary pneumococcal pneumonia, which should be further studied and validated in clinical trials. Due to the absence of a long-lasting immunity, re-infection with RSV throughout life is common. Thus, a possible perspective use could be a seasonal application of a nasal probiotic spray to boost respiratory innate immunity in immunocompetent subjects.

## Introduction

*Corynebacterium pseudodiphtheriticum* is a non-lipophilic, non-fermentative, urease- and nitrate-positive Gram-positive bacterium with variable shape (rods and cocci), which is found as a member of the normal microbiota of the human skin and upper respiratory tract (Ahmed et al., 1995; Burke et al., 1997; Bittar et al., 2010; Olender and Niemcewicz, 2010). Although there are clinical case reports pointing out at this bacterium as an opportunistic pathogen, it was suggested that *C. pseudodiphtheriticum*, being a natural member of the normal microbiota of nares and throat, may be potentially used as a probiotic for nasal application. In this regard, it has been demonstrated that *C. pseudodiphtheriticum* 090104 “Sokolov” is able to reduce *Staphylococcus aureus* colonization in humans (Uehara et al., 2000). There is also an inverse association between *S. aureus* and corynebacteria suggesting microbial competition during colonization (Liu C. M. et al., 2015). Controversially, some probiotic properties such as adherence to epithelial cells, biofilm formation, certain degree of immune stimulation, competition for nutrients, and adhesion sites are also shared by pathogens. For instance, some of the most known and used species of probiotic bacteria such as *Lactobacillus rhamnosus, L. plantarum, Enterococcus faecium, E. faecalis*, and even *E. coli* strain Nissle were also reported, though exceptionally, in clinical case reports. In any case, it has been established that security aspects such as antibiotic resistance or the presence of virulence factors are strain specific. Therefore, in order to propose *C. pseudodiphtheriticum* 090104 as a probiotic strain detailed studies evaluating both its functional properties and security aspects are necessary.

Respiratory syncytial virus (RSV) is a main respiratory pathogen responsible of bronchiolitis and pneumonia causing high morbidity and mortality in children under 3 years old. Currently, there are no available vaccines to prevent RSV infections or specific therapeutic treatments. Both viral and host factors are involved in disease severity. RSV cytopathogenicity is limited, but it elicits a strong immune response, which may result in tissue injury, loss of function and even death (Rutigliano and Graham, 2004; Bem et al., 2011). When exacerbated, immune response turns pathological, and in the case of RSV infection is characterized by high levels of pro-inflammatory chemokines and cytokines such as interleukin (IL)-6, IL-8, macrophage inflammatory protein (MIP)-1, tumor necrosis factor (TNF)-α, monocyte chemotactic protein (MCP)-1, and RANTES. At the very early stages of RSV infection, these pro-inflammatory factors participate in virus clearance, but their continuous production leads to increased injury (Rutigliano and Graham, 2004; Bem et al., 2011). In addition, secondary bacterial pneumonia is an important complication responsible for high morbidity and mortality of respiratory infections in infants and children (Liu et al., 2012; Bosch et al., 2013; Liu L. et al., 2015). The prevalence of bacteremia in children with RSV infection reported in the literature is low, ranging between 0.6 and 1.1% when conventional cultures were performed (Levine et al., 2004; Hishiki et al., 2011). However, a recent study showed that one out of every 10 previously healthy children hospitalized due to RSV had bacteremia, and these patients experienced a more severe disease (Cebey-Lopez et al., 2016). The rates of concurrent bacteremia was 10 times higher (10.6%) when molecular methods were applied. These findings are of importance because studies in clinical trials (Weinberger et al., 2013; Cebey-Lopez et al., 2016) and animal models of RSV-*Streptococcus pneumoniae* superinfection (Hament et al., 2005; Smith et al., 2014) showed that enhanced lung injuries and elevated levels of bacteremia are critical factors that determine the severity of infection and the rate of mortality.

Finding alternative strategies for the prevention of primary viral respiratory infections and secondary pneumococcal pneumonia in populations at risk is mandatory. In this regard, we have evaluated the possibility of administering probiotics to enhance the natural respiratory host defenses. In previous work, we showed that oral or nasal application of the probiotic strain *L. rhamnosus* CRL1505 protects adult and infant mice from RSV lethal challenge (Chiba et al., 2013; Tomosada et al., 2013). The main mechanism responsible for protection induced by the CRL1505 strain was enhancement of the mucosal antiviral innate immunity and the reduction of immunopathology. We hypothesized that a commensal bacterium from the respiratory tract such as *C. pseudodiphtheriticum* 090104 would modulate the respiratory antiviral innate immune response and beneficially influence the resistance to secondary bacterial infections. Therefore, in the present study we investigated how the exposure of infant mice to nasal priming with viable or non-viable human isolate *C. pseudodiphtheriticum* 090104 influences the respiratory innate immune response triggered by Toll-like receptor (TLR)-3 activation, the susceptibility to primary RSV infection, and the resistance to secondary *S. pneumoniae* pneumonia.

## Materials and methods

### Microorganisms

*Corynebacterium pseudodiphtheriticum* 090104 was cultured in trypticase soy broth and harvested by centrifugation at 3,000 × g for 10 min, washed three times with sterile 0.01 M phosphate buffer saline (PBS, pH 7.2), and resuspended in sterile PBS. Non-viable *C. pseudodiphtheriticum* 090104 was obtained as described previously (Tomosada et al., 2013). Briefly, bacteria were killed by tyndallization in a water bath at 80°C for 30 min and the lack of bacterial growth was confirmed by plating on to soy broth agar plates.

### Safety studies

In order to evaluate the susceptibility to antimicrobials of *C. pseudodiphtheriticum* 090104, the broth microdilution method was followed as recommended by the CLSI for infrequently isolated or fastidious bacteria (M45-A, CLSI-2006). The antimicrobial agents were penicillin (PEN), cefotaxime (CTX), ceftriaxone (CRO), meropenem (MER), vancomycin (VAN), gentamicin (GEN), ciprofloxacin (CIP), erythromicin (ERY), tetracycline (TET). MIC results were interpreted following CLSI guidelines (M45-A, CLSI-2006). Evaluation of bacterial translocation in infant mice was monitored by determining the presence of *C. pseudodiphtheriticum* lung, blood and spleen samples.

### Animals and feeding procedures

Female 3-week-old BALB/c mice were obtained from the closed colony kept at CERELA (San Miguel de Tucumán, Argentina) or Tohoku University (Sendai, Japan). They were housed in plastic cages at room temperature. Mice were housed individually during the experiments. Viable and non-viable *C. pseudodiphtheriticum* 090104 were nasally administered to mice for 5 consecutive days at a dose of 10^8^ cells/mouse/day in 50 μl of PBS. All groups were fed a conventional balanced diet *ad libitum*. This study was carried out in strict accordance with the recommendations in the Guide for the Care and Use of Laboratory Animals of the Guidelines for Animal Experimentation of CERELA and all efforts were made to minimize suffering. The Institutional Animal Welfare Committee of CERELA reviewed and approved the protocols used in this study.

### Intranasal administration of poly(I:C)

Administration of the viral pathogen molecular pattern poly(I:C) was performed on day 6, after a 5 days treatment with viable or non-viable *C. pseudodiphtheriticum* 090104. Mice were lightly anesthetized and 100 μl of PBS, containing 250 μg poly(I:C) (equivalent to 10 mg/kg body weight), was administered dropwise, via the nares (Tomosada et al., 2013). Control animals received 100 μl of PBS. Mice received three doses of poly(I:C) or PBS with 24 h rest period between each administration.

### Respiratory syncytial virus primary infection

Human RSV strain A2 was grown in Vero cells as described by Murawski et al. (2009). Briefly, Vero cells were infected with RSV at a multiplicity of infection (MOI) of 1 in 5 ml of Dulbecco's modified Eagle's medium (DMEM). Cells were infected for 2.5 h at 37°C and 5% CO_2_. After infection, 7 ml of DMEM with 10% FBS (Sigma, Tokyo, Japan), 0.1% penicillin–streptomycin (Pen/Strep) (Sigma, Tokyo, Japan), and 0.001% ciprofloxacin (Bayer) were added to the flask and further incubated. When extensive syncytia formed, cells were scraped from the flask and sonicated three times, 5 s per pulse, at 25 W on ice. Cell lysates were centrifuge at 700 × *g* for 10 min at 4°C. Cell-free virus stocks were stored in 30% sucrose at −80°C. Uninfected cells were treated identically to generate a virus- and cell-free supernatant control. Mice were slightly anesthetized and intranasally challenged with 2.4 × 10^6^ PFU RSV strain A2 or an equivalent volume of Vero cell lysate on day 6 after viable or non-viable *C. pseudodiphtheriticum* 090104 treatment.

Intact lung tissue was removed and stored in 30% sucrose for plaque assays. RSV immune-plaque assay was done as previously explained (Chiba et al., 2013). Briefly, lungs were homogenized using a pellet pestle and centrifuged at 2,600 × *g* for 10 min at 4°C. Twenty-four-well tissue culture plates were seeded with 1.5 × 10^5^ Vero cells/well in DMEM (10% FBS, 0.1% Pen/Strep, and 0.001% ciprofloxacin) and virus plaques assays were performed in triplicate. Plates were incubated at 37°C and 5% CO_2_ for 2.5 h. After incubation, supernatant was removed, and 1 ml of fresh DMEM medium supplemented with 10% FBS, 0.1% Pen/Strep, and 0.001% ciprofloxacin was overlaid on monolayers. When extensive syncytia developed, the overlay was removed and monolayers were fixed with ice-cold acetone:methanol (60:40). Primary RSV anti-F (clones131-2A; Chemicon) and anti-G (Mouse monoclonal [8C5 (9B6)] to RSV glycoprotein, Abcam) antibodies were added to wells for 2 h, followed by secondary horseradish peroxidase anti-mouse immunoglobulin antibody (Anti-mouse IgG, HRP-linked Antibody #7076, Cell signaling Technology) for 1 h. Plates washed twice with PBS containing 0.5% Tween 20 (Sigma) after each antibody incubation step. Individual plaques were developed using a DAB substrate kit (ab64238, Abcam) following manufacture's instructions. Results for immune-plaque assay were expressed as log_10_ PFU/g of lung.

### *Streptococcus pneumoniae* secondary infection

*Streptococcus pneumoniae* serotype 6B (ANLIS, Argentina) was obtained from the respiratory tract of a patient from Hospital del Niño Jesús, Tucumán, Argentina. Pneumococci were grown on blood agar for 18 h. Colonies were suspended in Todd Hewitt broth (Oxoid), incubated overnight at 37°C, harvested and washed with sterile PBS. Cell density was adjusted to 4 × 10^7^ CFU/ml. Challenge with pneumococci was performed 5 days after the last administration of poly(I:C) or RSV challenge. Viable or non-viable *C. pseudodiphtheriticum*-treated as well as control infant mice were challenged intranasally with the pathogen by dripping 25 μl of inoculums containing 10^3^ CFU (log phase) in PBS into each nostril.

Treated and control mice were sacrificed 2 days after *S. pneumoniae* infection. Lungs were excised, weighed and homogenized in sterile peptone water. Homogenates were diluted appropriately, plated in duplicate on blood agar and incubated for 18 h at 37°C. *Streptococcus pneumoniae* was identified by standard techniques and the results were expressed as log of CFU/g of lung. Bacteremia was monitored in blood samples obtained by cardiac puncture which were plated on blood agar. Results were reported as negative or positive hemocultures.

### Cytokine concentrations in broncho-alveolar lavages (BAL)

BAL samples were obtained as described previously (Villena et al., 2005) by performing lavages of lungs with sterile PBS. After centrifugation, cell-free supernatants were kept at −70°C. Tumor necrosis factor (TNF)-α, interferon (IFN)-γ, IFN-β, IFN-α, interleukin (IL)-6, and IL-10 concentrations in serum and BAL were measured by enzyme-linked immunosorbent assay (ELISA) following the manufacturer's recommendations (R&D Systems, MN, USA) (Salva et al., 2011).

### Lung cell suspensions

Single lung cells were prepared using the previously described method (Villena et al., 2012). Briefly, mice were anesthetized and lungs were removed, finely minced and incubated for 90 min with 300 U of collagenase (Yakult Honsha Co., Tokyo, Japan) in 15 ml of RPMI 1640 medium (Sigma, Tokyo, Japan). After removal of debris, erythrocytes were depleted by hypotonic lysis. The cells were washed with RPMI medium supplemented with 0.1% Pen/Strep and suspended in a medium supplemented with 10% heat-inactivated fetal calf serum (FCS). Cells were counted using Trypan Blue and adjusted to 5 × 10^6^ cells/ml.

### Flow cytometry studies

Single lung cells from mice were prepared as previously described (Villena et al., 2012; Zelaya et al., 2014, 2015). Lungs were removed, finely minced and incubated for 90 min with 300 U of collagenase (Yakult Honsha Co., Tokyo, Japan) in 15 ml of RPMI 1640 medium (Sigma, Tokyo, Japan). To dissociate the tissue into single cells, collagenase-treated minced lungs were gently tapped into a plastic dish. After removal of debris, erythrocytes were depleted by hypotonic lysis. The cells were washed with RPMI medium supplemented with 100 U/ml of penicillin and 100 mg/ml of streptomycin and then resuspended in a medium supplemented with 10% heat-inactivated fetal calf serum (FCS). Cells were counted using Trypan Blue exclusion and then resuspended at 5 × 10^6^ cells/ml.

Lung cell suspensions were pre-incubated with anti-mouse CD32/CD16 monoclonal antibody (Fc block) for 15 min at 4°C. Cells were incubated in the antibody mixes for 30 min at 4°C and washed with FACS buffer. Then, cells were stained with fluorochrome-conjugated antibodies against CD3, CD4, CD8, CD11c, CD11b, CD103, MHC-II, IFN-γ, IL-10, sialic acid-binding immunoglobulin-like lectin F (SiglecF) (BD Bioscience), IFN-β, and CD45 (eBioscience). Cells were then acquired on a BD FACSCalibur™ flow cytometer (BD Biosciences) and data were analyzed with FlowJo software (TreeStar). The total number of cells in each population was determined by multiplying the percentages of subsets within a series of marker negative or positive gates by the total cell number determined for each tissue (Villena et al., 2012; Zelaya et al., 2014, 2015).

### Lung tissue injury studies

To measure increased permeability of the bronchoalveolar–capillarity barrier, we quantified albumin and protein content in cell-free BAL. Furthermore, lactate dehydrogenase (LDH) activity was quantified as an indicator of general cytotoxicity (Villena et al., 2012; Zelaya et al., 2014, 2015). Lung wet:dry weight ratio was determined as described before. Briefly, mice were euthanized and exsanguinated; lungs were removed, weighed (wet weight), dried at 55°C for 7 days, and weighed again (dry weight). The wet:dry weight ratio is a measure of intrapulmonary fluid accumulation. Histopathological examination was also performed in order to evaluate tissue damage during respiratory superinfection. Lungs were aseptically removed, fixed in 4% formalin and embedded in histowax (Leica Microsystems). Histopathological assessment was performed on five-micron tissue sections stained with hematoxylin-eosin.

### Statistical analysis

Experiments were performed in triplicate and results were expressed as mean ± standard deviation (SD). Five to six animals were used in each replicate per experimental group. Normal distributed data were tested by 2-way ANOVA was used. Tukey's test (for pairwise comparisons of the means) or the Fisher's least significant difference (LSD) test (for multi-comparison) were used to evaluate the differences between the groups. Differences were considered significant at *p* < 0.05.

## Results

### Influence of viable and non-viable *C. pseudodiphtheriticum* on respiratory immune response

**Figure 1:**
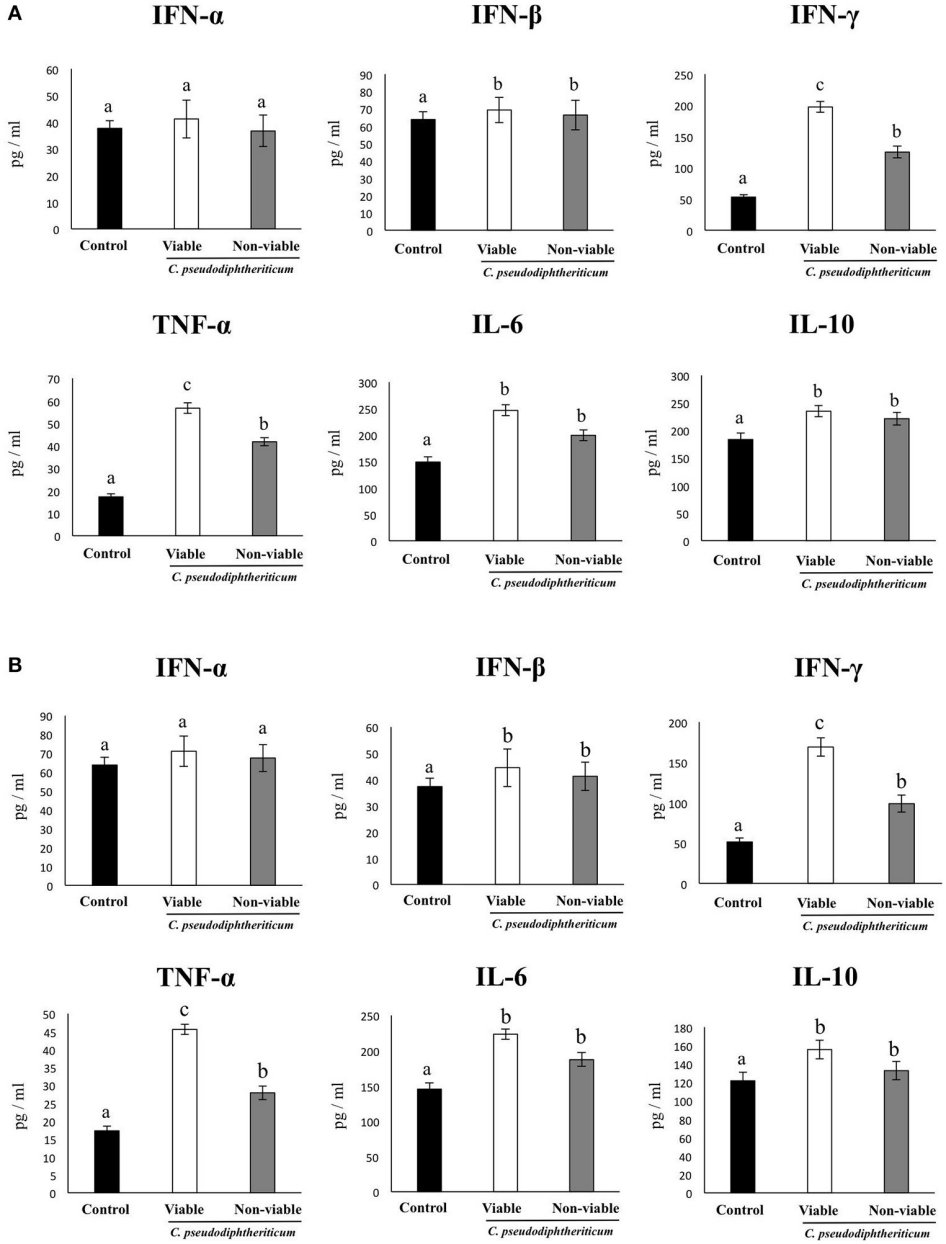
Effect of *Corynebacterium pseudodiphtheriticum* strain 090104 on respiratory and blood cytokines. Viable or non-viable *C. pseudodiphtheriticum* were nasally administered to infant mice during five consecutive days. Non-treated infant mice were used as controls. Levels of tumor necrosis factor (TNF)-α, interferon (IFN)-α, IFN-β, IFN-γ, interleukin (IL)-6, and IL-10 were determined in broncho-alveolar lavages (BAL) **(A)** and serum **(B)**. Experiments were performed with 5–6 mice per group. The results represent data from three independent experiments. Values for bars with different letters were significantly different (*P* < 0.05). Values for bars with shared letters do not differ significantly.

In order to determine whether viable or non-viable *C. pseudodiphtheriticum* could modulate mucosal immune responses in the respiratory tract, we quantified key cytokines in broncho-alveolar lavages (BAL) and serum samples as well as immune cell populations in lungs. Both treatments elicited an increase in the levels of the proinflammatory cytokines TNF-α and IL-6 in serum and BAL (Figure 1). Furthermore, *C. pseudodiphtheriticum* induced a significant increase in the production of IFN-γ and a slight but still significant enhancement of IFN-β, while no effect was observed in IFN-α levels. The immunoregulatory cytokine IL-10 was also detected at higher concentrations in viable or non-viable *C. pseudodiphtheriticum*-treated infant mice than in control mice without bacterial stimulation (Figure 1). In general, the effect of the live bacterium was stronger than the elicited by non-viable *C. pseudodiphtheriticum*. In addition, the changes in cytokine levels were more pronounced in BAL (Figure 1A) than in serum samples (Figure 1B).

**Figure 2:**
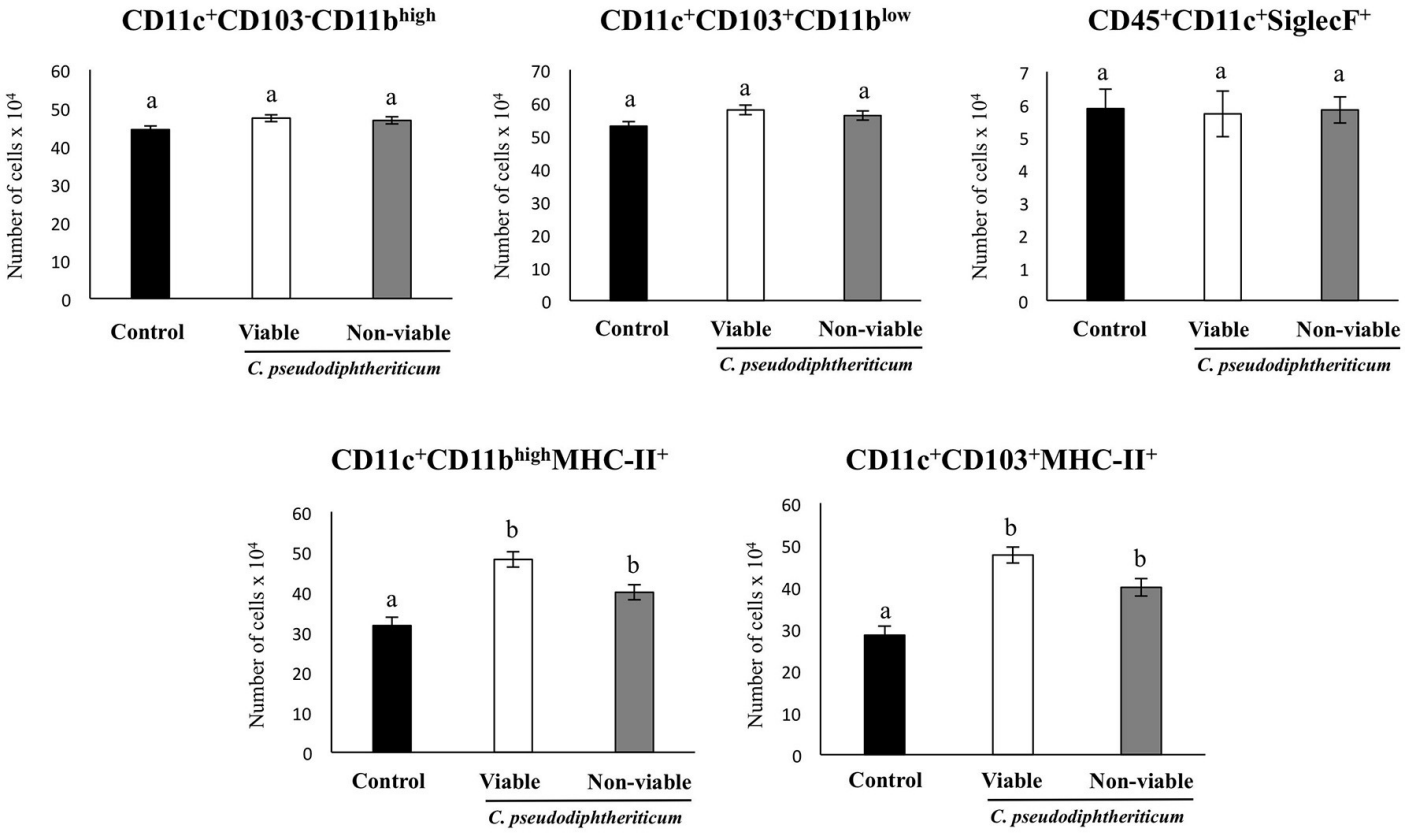
Effect of *Corynebacterium pseudodiphtheriticum* strain 090104 on lung immune cell populations. Viable or non-viable *C. pseudodiphtheriticum* were nasally administered to infant mice during five consecutive days. Non-treated infant mice were used as controls. The numbers of lung antigen presenting cells including MHC-II^+^CD11c^+^CD11b^low^CD103^+^ and MHC-II^+^CD11c^+^CD11b^high^CD103^−^ dendritic cells, and CD45^+^MHC-II^−^CD11c^+^SiglecF^+^ alveolar macrophages were determined by flow cytometry. Experiments were performed with 5–6 mice per group. The results represent data from three independent experiments. Values for bars with different letters were significantly different (*P* < 0.05). Values for bars with shared letters do not differ significantly.

**Figure 3:**
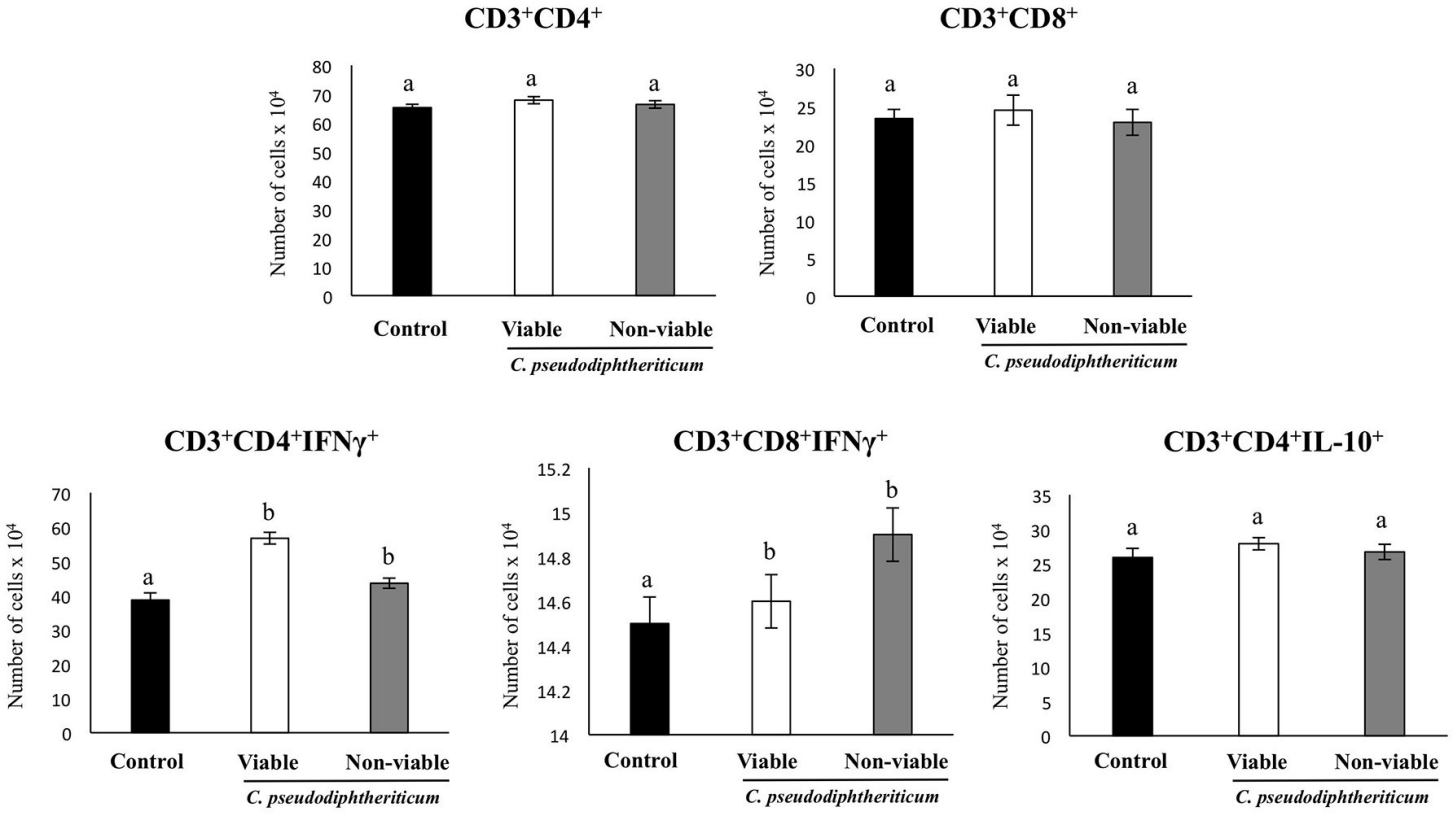
Effect of *Corynebacterium pseudodiphtheriticum* strain 090104 on lung immune cell populations. Viable or non-viable *C. pseudodiphtheriticum* were nasally administered to infant mice during five consecutive days. Non-treated infant mice were used as controls. The numbers of lung T cells including CD3^+^CD4^+^IFN-γ^+^, CD3^+^CD4^+^IL-10^+^, and CD3^+^CD8^+^IFN-γ^+^ T lymphocytes were determined by flow cytometry. Experiments were performed with 5–6 mice per group. The results represent data from three independent experiments. Values for bars with different letters were significantly different (*P* < 0.05). Values for bars with shared letters do not differ significantly.

Flow cytometry indicated that there were no significant differences between the experimental groups when the numbers of lung antigen presenting cells including dendritic cells (CD11c^+^CD103-CD11b^high^ and CD11c^+^CD103^+^CD11b^low^ cells) and alveolar macrophages (CD45^+^CD11c^+^SiglecF^+^ cells) were compared (Figure 2). However, activated lung antigen presenting cells differed in mice nasally treated with *C. pseudodiphtheriticum* (Figure 2). For instance, CD11c^+^CD103^+^MHCII^+^ and CD11c^+^CD11b^high^MHCII^+^ populations were significantly greater (*p* < 0.05) in viable or non-viable *C. pseudodiphtheriticum*-treated infant mice than in control mice. In both cases, live bacteria had a stronger influence than non-viable *C. pseudodiphtheriticum* in antigen presenting cells activation. No differences were observed between the groups when total counts of lung CD3^+^CD4^+^ and CD3^+^CD8^+^ T cells were compared (Figure 3). Nevertheless, *C. pseudodiphtheriticum* differentially modulated specific IFN-γ producing populations of CD3^+^CD4^+^ and CD3^+^CD8^+^ lymphocytes. Viable bacteria significantly induced higher numbers of IFN-γ specific CD3^+^CD4^+^ T cells, whereas heat-killed *C. pseudodiphtheriticum* increased IFN-γ specific CD3^+^CD8^+^ cells (Figure 3). A slight but not significant increase of lung CD3^+^CD4^+^IL-10^+^ T cells was observed in *C. pseudodiphtheriticum*-treated mice.

### *Corynebacterium pseudodiphtheriticum* modulates immune response triggered by poly(I:C) and reduces lung injury

**Figure 4:**
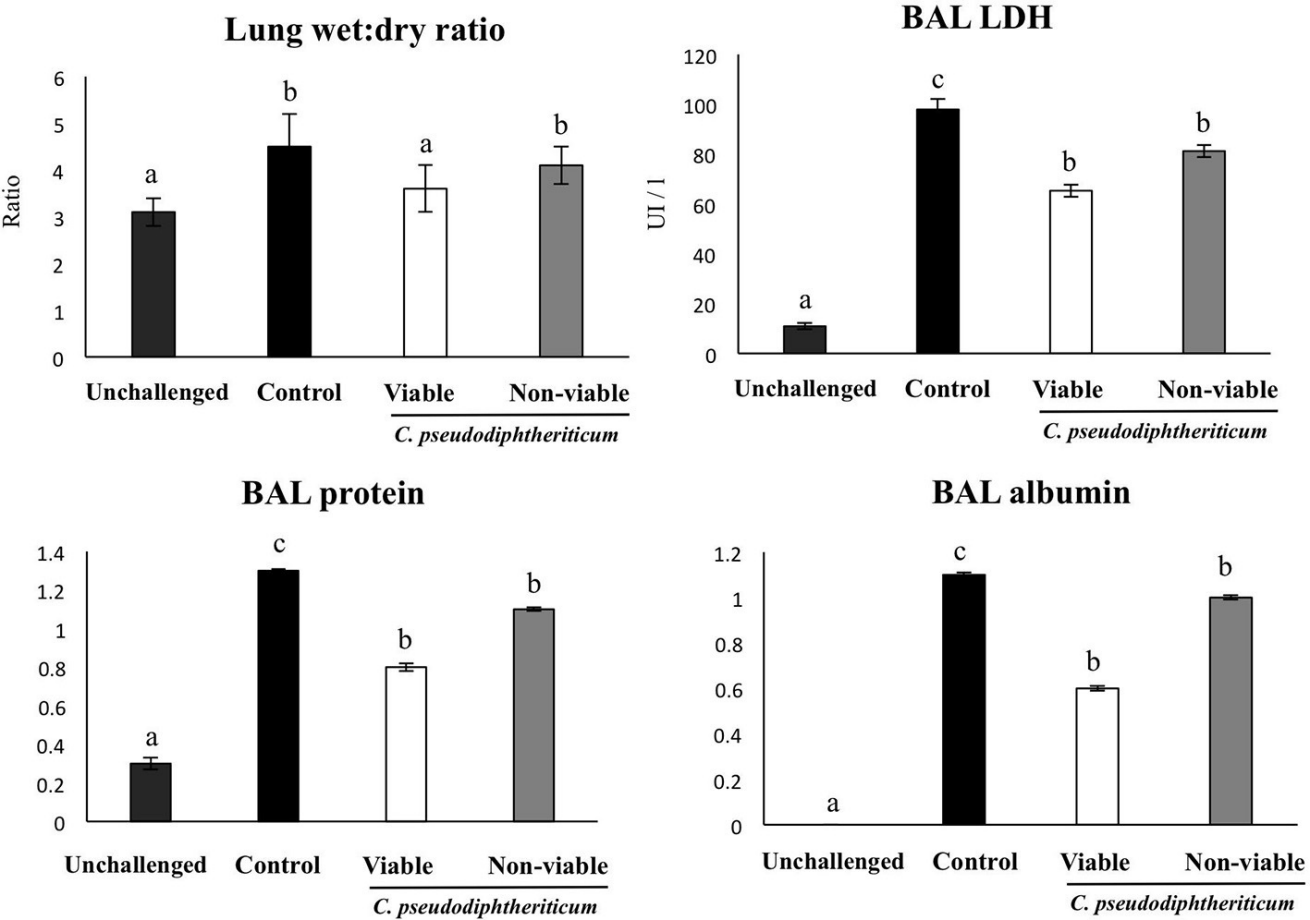
Effect of *Corynebacterium pseudodiphtheriticum* strain 090104 on lung tissue damage induced by the nasal administration of the viral pathogen-associated molecular pattern poly(I:C). Infant mice were nasally primed with viable or non-viable *C. pseudodiphtheriticum* during five consecutive days and then challenged with three once-daily doses of poly(I:C). Non-treated infant mice challenged with poly(I:C) were used as controls. Two days after the last poly(I:C) administration lung wet:dry weight ratio, lactate dehydrogenase (LDH) activity and, albumin and protein concentrations in broncho-alveolar lavages (BAL) were determined. Experiments were performed with 5–6 mice per group. The results represent data from three independent experiments. Values for bars with different letters were significantly different (*P* < 0.05). Values for bars with shared letters do not differ significantly.

After treatment with viable or non-viable *C. pseudodiphtheriticum*, mice were nasally challenged for three consecutive days with poly(I:C). In order to evaluate the extent of lung injury induced by TLR3-triggered inflammation (Figure 4), we determined total protein and albumin concentrations, and LDH activity in BAL 2 days after challenge. In addition, we also determined the magnitude of edema by calculating the lung wet:dry ratio. Administration of poly(I:C) resulted in water retention due to the inflammatory response in the lungs in comparison with unchallenged mice. Viable *C. pseudodiphtheriticum* somehow prevented lungs from retaining fluids as it is shown in Figure 4 reaching levels close to the unchallenged lung wet:dry ratios. Other lung injury parameters, such as protein content, albumin and LDH activity were also significantly (*p* < 0.05) reduced when mice were previously treated with viable or non-viable *C. pseudodiphtheriticum*, being the treatment with live bacteria more effective than heat-killed bacteria.

**Figure 5:**
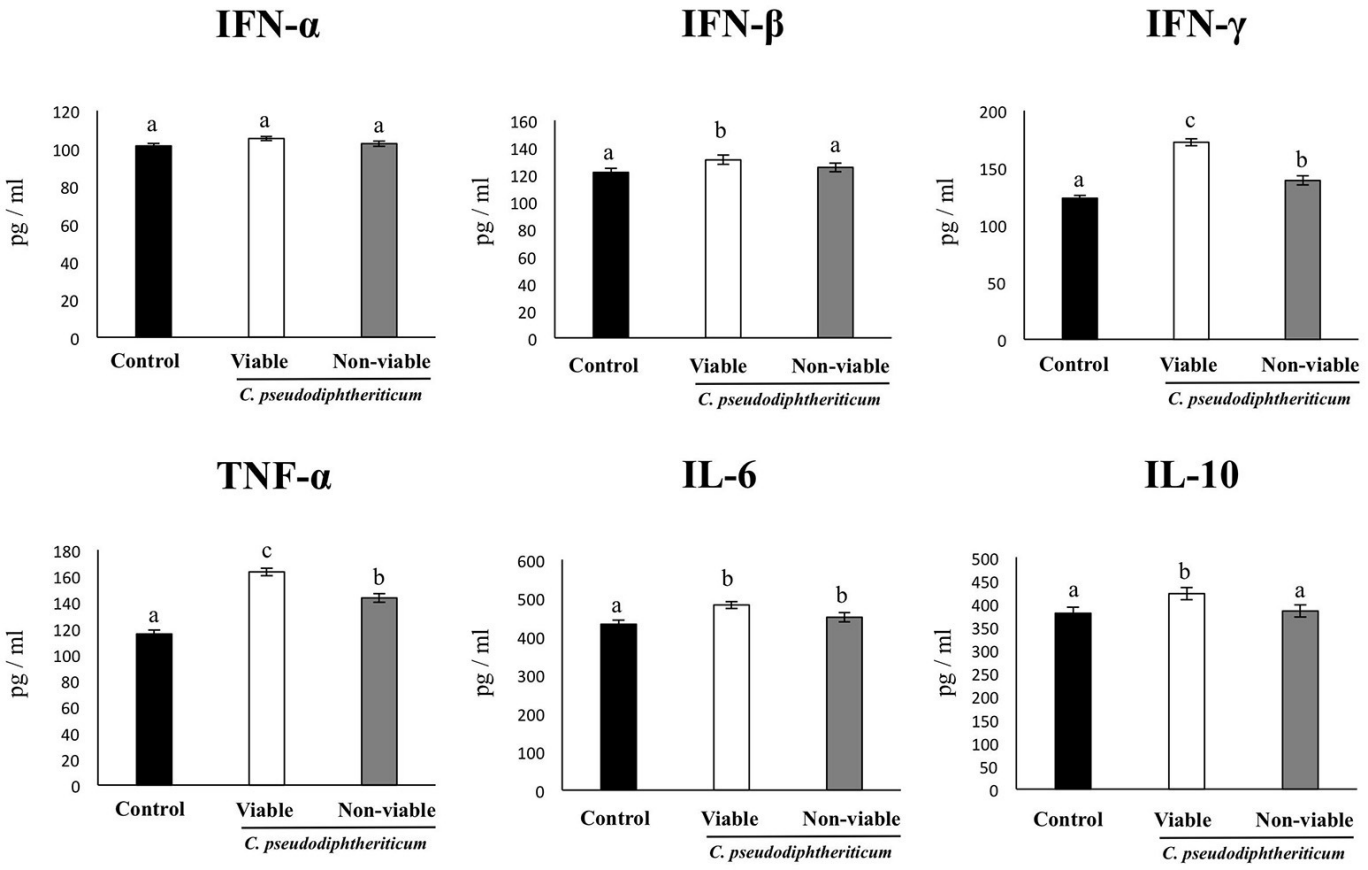
Effect of *Corynebacterium pseudodiphtheriticum* strain 090104 on respiratory cytokines after the nasal administration of the viral pathogen-associated molecular pattern poly(I:C). Infant mice were nasally primed with viable or non-viable *C. pseudodiphtheriticum* during five consecutive days and then challenged with three once-daily doses of poly(I:C). Non-treated infant mice challenged with poly(I:C) were used as controls. Two days after the last poly(I:C) administration the levels of tumor necrosis factor (TNF)-α, interferon (IFN)-α, IFN-β, IFN-γ, interleukin (IL)-6, and IL-10 were determined in broncho-alveolar lavages (BAL). Experiments were performed with 5–6 mice per group. The results represent data from three independent experiments. Values for bars with different letters were significantly different (*P* < 0.05). Values for bars with shared letters do not differ significantly.

**Figure 6:**
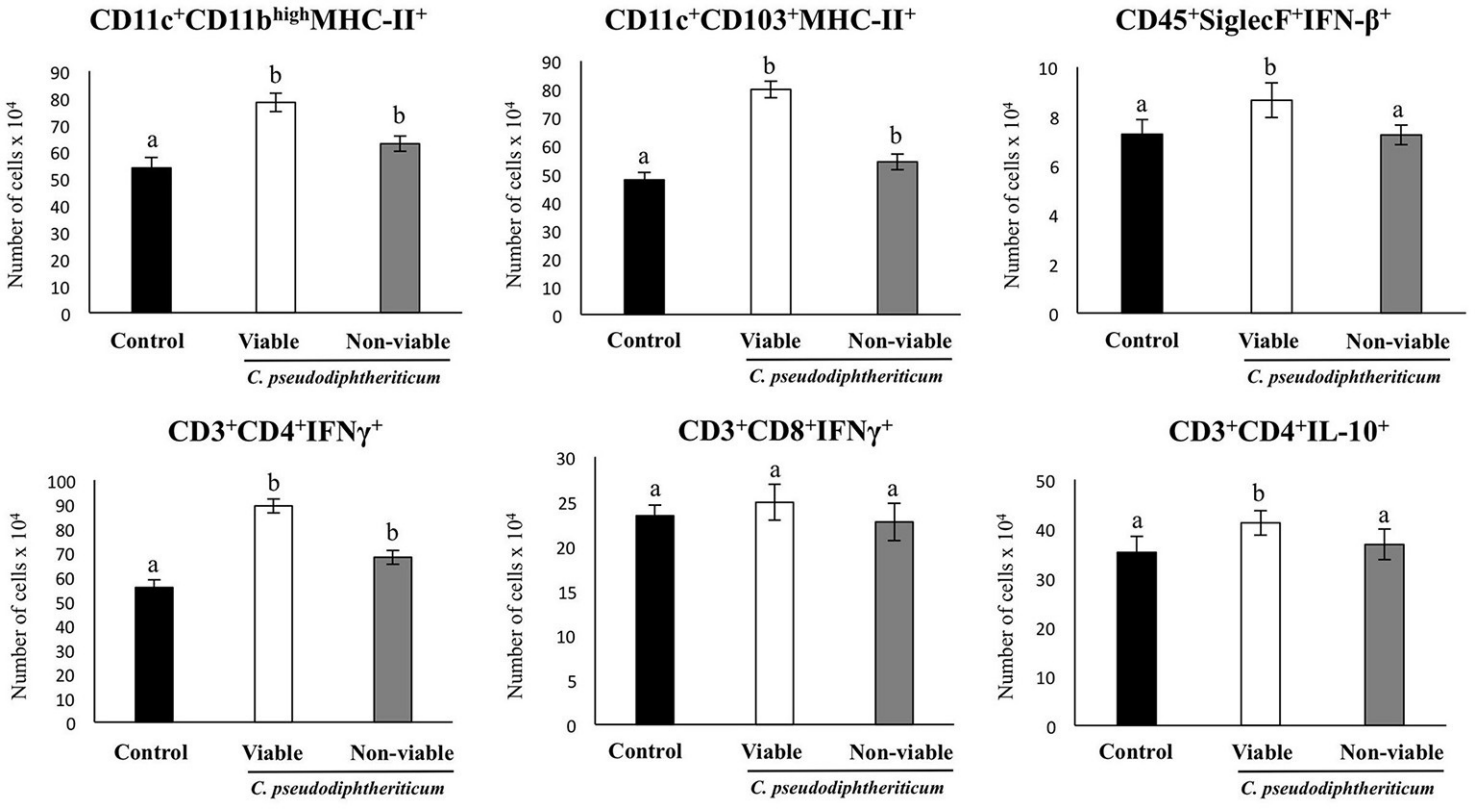
Effect of *Corynebacterium pseudodiphtheriticum* strain 090104 on respiratory immune cell populations after the nasal administration of the viral pathogen-associated molecular pattern poly(I:C). Infant mice were nasally primed with viable or non-viable *C. pseudodiphtheriticum* during five consecutive days and then challenged with three once-daily doses of poly(I:C). Non-treated infant mice challenged with poly(I:C) were used as controls. Two days after the last poly(I:C) administration the numbers of lung T cells including CD3^+^CD4^+^IFN-γ^+^, CD3^+^CD4^+^IL-10^+^, and CD3^+^CD8^+^IFN-γ^+^ T lymphocytes, as well as antigen presenting cells including MHC-II^+^CD11c^+^CD11b^low^CD103^+^ and MHC-II^+^CD11c^+^CD11b^high^CD103^−^ dendritic cells, and CD45^+^MHC-II^−^CD11c^+^SiglecF^+^ alveolar macrophages were determined by flow cytometry. Experiments were performed with 5–6 mice per group. The results represent data from three independent experiments. Values for bars with different letters were significantly different (*P* < 0.05). Values for bars with shared letters do not differ significantly.

As we have described previously (Tomosada et al., 2013), nasal challenge with poly(I:C) increased the levels of proinflammatory cytokines and IL-10 (Figure 5), and immune cells (Figure 6) in the respiratory tract when compared to basal levels. The nasal pretreatments of mice with viable or non-viable *C. pseudodiphtheriticum* differentially modulated the profiles of cytokines measured in BAL. IFN-γ was the most influenced cytokine, showing a significant increase in mice treated with live bacteria in comparison to untreated control mice challenged with poly(I:C), whereas non-viable bacteria slightly increased the levels of IFN-γ (Figure 5). To a lesser extent, TNF-α, IL-6, IFN-β, and IL-10 concentrations in BAL were also higher in viable *C. pseudodiphtheriticum*-treated mice. In contrast, only TNF-α and IL-6 were modulated by non-viable bacteria.

The numbers of lung CD11c^+^CD103^+^MHCII^+^ and CD11c^+^CD11b^high^MHCII^+^ dendritic cells were significantly greater (*p* < 0.05) in viable or non-viable *C. pseudodiphtheriticum*-treated infant mice than in control mice (Figure 6). In addition, higher numbers of CD45^+^SiglecF^+^IFN-β^+^ alveolar macrophages and CD3^+^CD4^+^IL-10^+^ T cells were observed when mice were previously treated with viable *C. pseudodiphtheriticum* in comparison to control mice (Figure 6). In contrast, non-viable bacteria did not produce significant changes in the numbers of these immune cells populations. CD3^+^CD4^+^IFNγ^+^ lymphocyte counts increased in mice which received viable or non-viable *C. pseudodiphtheriticum* before poly(I:C) challenge, being this effect stronger with the viable bacteria (Figure 6).

### Viable *C. pseudodiphtheriticum* increases resistance to primary RSV infection

**Figure 7:**
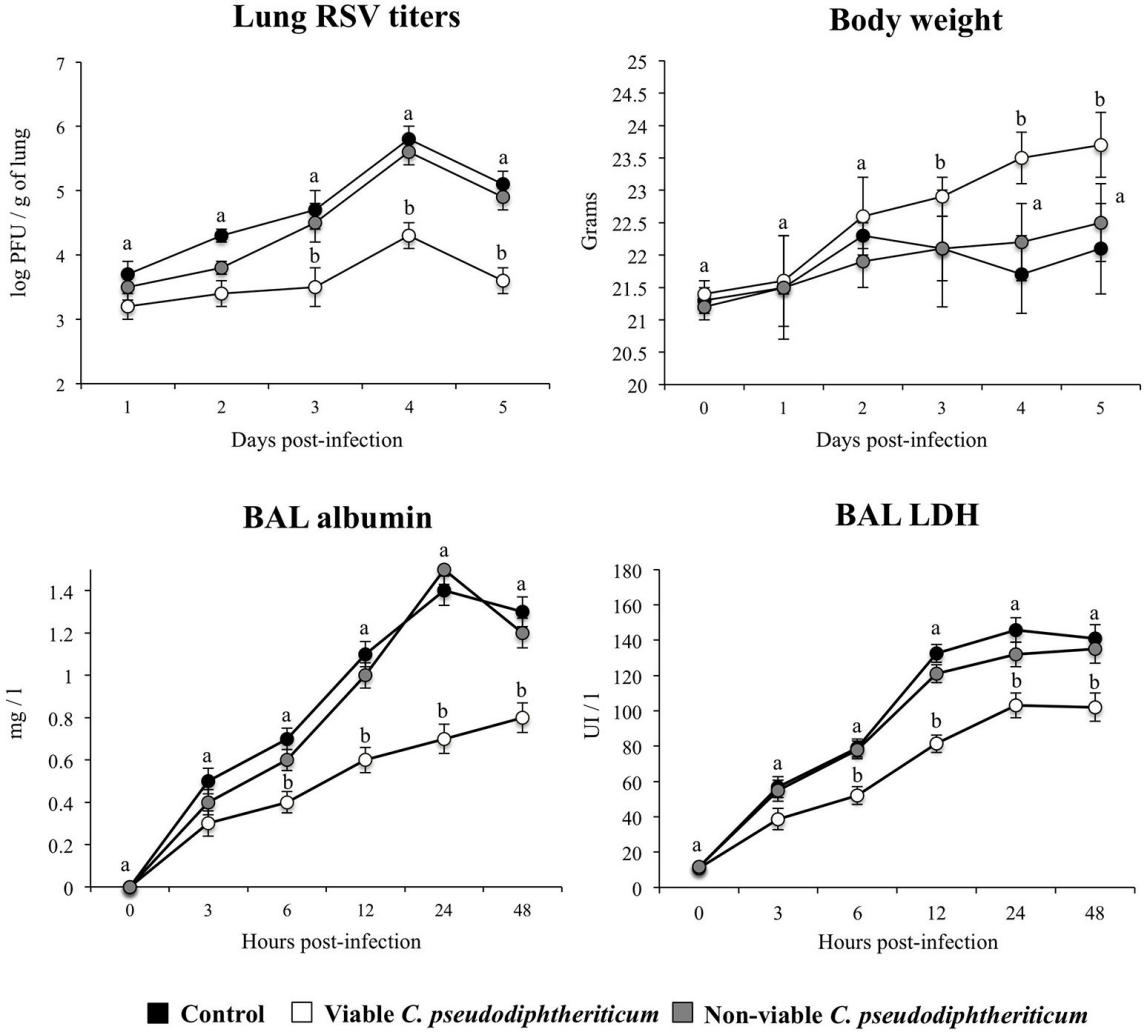
Effect of *Corynebacterium pseudodiphtheriticum* strain 090104 on the resistance to primary Respiratory Syncytial Virus (RSV) infection. Infant mice were nasally primed with viable or non-viable *C. pseudodiphtheriticum* during five consecutive days and then challenged with RSV. Non-treated infant mice challenged with the viral pathogen were used as controls. Lung RSV titers, changes in body weight, and lactate dehydrogenase (LDH) activity and albumin concentrations in broncho-alveolar lavages (BAL) were evaluated on different time points after the viral challenge. Experiments were performed with 5–6 mice per group per each time point. The results represent data from three independent experiments. Values for each time point with different letters were significantly different (*P* < 0.05). Values for each time point with shared letters do not differ significantly.

Nasal administration of viable *C. pseudodiphtheriticum* improved health state of infant mice infected with RSV as reflected by the increase in body weight during the studied period (Figure 7). Mice treated with non-viable bacteria did not show the improvement of body weight. Virus load was significantly lower in mice treated with viable *C. pseudodiphtheriticum* when compared to those receiving heat-killed bacteria or controls (Figure 7). The markers of lung tissue damage in RSV-infected mice showed that the viral infection induced a significant cellular damage and alveolar-capillary barrier alterations (Figure 7). Both, BAL LDH and albumin concentrations were significantly lower in infant mice previously treated with viable *C. pseudodiphtheriticum* than in RSV-challenged controls or mice receiving heat-killed bacteria (Figure 7).

### Viable *C. pseudodiphtheriticum* increase resistance to secondary pneumococcal infection

**Figure 8:**
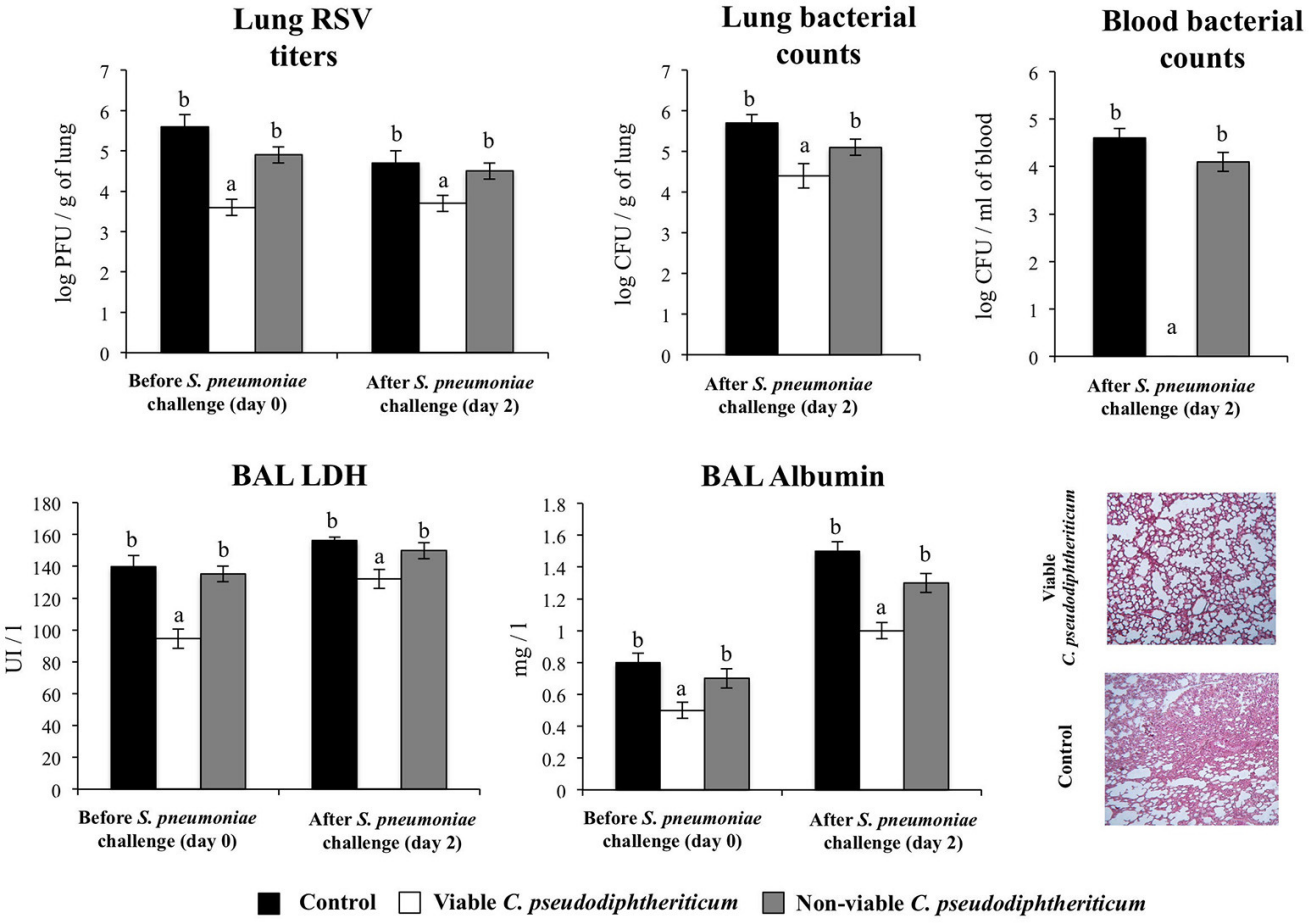
Effect of *Corynebacterium pseudodiphtheriticum* strain 090104 on the resistance to secondary pneumococcal pneumonia after the primary infection with Respiratory Syncytial Virus (RSV). Infant mice were nasally primed with viable or non-viable *C. pseudodiphtheriticum* during five consecutive days, challenged with RSV and, infected with *Streptococcus pneumoniae* 5 days after the viral infection. Non-treated infant mice infected with RSV and challenged with *S. pneumoniae* were used as controls. Lung RSV titers, lung bacterial cells counts, lactate dehydrogenase (LDH) activity and albumin concentrations in broncho-alveolar lavages (BAL), and lung histology were determined on day 2 post-pneumococcal challenge. Lungs histological examination was performed with hematoxylin and eosin stained light micrographs, original magnification ×40. Experiments were performed with 5–6 mice per group. The results represent data from three independent experiments. Values for bars with different letters were significantly different (*P* < 0.05). Values for bars with shared letters do not differ significantly.

Finally, we addressed whether the nasal treatments with viable and non-viable *C. pseudodiphtheriticum* where able to increase the resistance of infant mice to secondary pneumococcal pneumonia. For that purpose, mice were nasally primed with viable or non-viable bacteria, infected with RSV, and 5 days after virus infection, they were challenged with *S. pneumoniae*. Pneumococcal colonization and bacteremia were evaluated on day 2 post-pneumococcal challenge. In addition, RSV titers as well as lung tissue damage were studied before (day 0) and after (day 2) infection with *S. pneumoniae*. RSV was detected in lungs of infected infant mice before and after pneumococcal infection (Figure 8). In addition, pneumococci were detected in lungs and blood of control infant mice (Figure 8). Viable *C. pseudodiphtheriticum* significantly reduced RSV titers as well as lung bacterial cell counts and prevented the dissemination of *S. pneumoniae* into the blood (Figure 8). No protective effect was observed for non-viable *C. pseudodiphtheriticum*. When lung injury was studied it was observed that the secondary pneumococcal pneumonia induced a significant increase of the BAL biochemical parameters that evaluate cellular damage and alveolar-capillary barrier alterations (Figure 8). Viable *C. pseudodiphtheriticum* significantly reduced pulmonary damage as demonstrated by the lower LDH and albumin content in BAL when compared to controls and non-viable bacteria-treated mice (Figure 8). Moreover, histological examination of lung of infected infant mice revealed severe inflammatory cell recruitment around alveoli and blood vessels, focal hemorrhage and a significant reduction of gas exchange spaces (Figure 8). Lung histology analysis of viable *C. pseudodiphtheriticum*-tretaed mice showed a significant reduction in the alterations of gas exchange spaces, hemorrhage and inflammatory cells infiltration (Figure 8) while mice treated with non-viable bacteria were not different from control animals (data not shown).

## Discussion

Respiratory viruses are an important cause of fatal pneumonia in children. They also predispose individuals to suffer bacterial infections by disrupting cells, releasing nutrients and reducing ciliary continuity and kinesia. Further, they alter the innate and adaptive immune systems, which may in turn help promoting bacterial infection (Hament et al., 2005; Smith et al., 2014). Therefore, there is a global need for controlling primary respiratory viral infections and secondary bacterial diseases, and beneficial microbes may offer an interesting alternative (Maragkoudakis et al., 2010; Villena et al., 2012; Chiba et al., 2013; Tomosada et al., 2013; Tada et al., 2016).

There is an increasing amount of evidence indicating that respiratory indigenous microbiota contributes to respiratory health by preventing the overgrowth of pathogens and the inflammation they cause (Pettigrew et al., 2012; Bosch et al., 2013). Commensal bacteria may exert health benefits by opposing to bacterial or viral pathogens directly by blocking adhesion sites and/or indirectly by modulating host immune responses in such a way that pathogen clearance is enhanced but inflammation is simultaneously better controlled (Uehara et al., 2000; Pettigrew et al., 2012; Kiryukhina et al., 2013; Liu C. M. et al., 2015). Therefore, respiratory commensal bacteria, if investigated in depth may be a source for developing next generation probiotic preparations for the improvement of respiratory health. In this study, we demonstrated that *C. pseudodiphtheriticum*, a typical commensal of the nasal human mucosa, is a candidate for enhancing respiratory immune responses and protecting against RSV and *S. pneumoniae* infections.

Although there are some safety concerns about *C. pseudodiphtheriticum* because of a few case reports indicating opportunistic infections by this bacterium, it is well accepted that both probiotic and safety properties are strain specific. Genomic analysis revealed the presence of a hemolysin protein in *C. pseudodiphtheriticum* 090104 genome with similar characteristics to the one found in *Bacillus cereus*, which could become a potential risk for health especially in immunocompromised individuals (Karlyshev and Melnikov, 2013). However, the 090104 strain was safe when used in the mouse model studied here, and in clinical trials in healthy volunteers performed before (Uehara et al., 2000; Kiryukhina et al., 2013; Liu C. M. et al., 2015). Our studies evaluating the potential translocation of *C. pseudodiphtheriticum* 090104 in infant mice after its nasal administration showed no adverse effects (data not shown). In addition, *C. pseudodiphtheriticum* 090104 shows resistance to β-lactam antibiotics and macrolides (data not shown) that are the most prevalent acquired antibiotic resistances described for this species. However, genome analysis did not evidence the presence of antibiotic resistance genes acquired by genetic horizontal transfer. Thus, our studies indicate that the use of this bacterium would be safe.

In previous studies, it has been shown that nasal application of *C. pseudodiphtheriticum* allowed the bacteria to colonize the nasal mucosa reducing *S. aureus* infection (Uehara et al., 2000; Kiryukhina et al., 2013; Liu C. M. et al., 2015). In line with these observations, we showed here for the first time that the nasal priming with *C. pseudodiphtheriticum* 090104 reduced RSV and *S. pneumoniae* colonization in infant mice. Moreover, this is the first study showing immunomodulatory properties for viable *C. pseudodiphtheriticum* as well as its capacity to enhance immunity against primary RSV and secondary pneumococcal pneumonia. According to our results, viable *C. pseudodiphtheriticum* was effective in reducing the burden of RSV infection as reflected by the lower viral load, improved body weight, and reduced pulmonary damage. The ameliorated pulmonary injury was related to the modulation of the inflammatory response that is known to be a main component of damage in RSV infections (Rutigliano and Graham, 2004; Bem et al., 2011; Cervantes-Ortiz et al., 2016). Main secreted proinflammatory cytokines in children coursing a natural RSV infection as well as in experimentally RSV inoculated mice are type I IFNs, TNF-α, IL-6, IL-8, MIP-1, RANTES, and MCP-1. Although these cytokines contribute to virus clearance in the early steps of infection, deregulated cytokine response leads to tissue injury (McNamara and Smyth, 2002). In our experiments, pre-treatment of infant mice with *C. pseudodiphtheriticum* enhanced the secretion of IFN-β, TNF-α, and IL-6 but at the same time, it also enhanced the production of IL-10 in response to RSV infection. It was proposed that the most prominent role of IL-10 is its contribution to restrict inflammation during RSV infection, which consequently lowers injury (Stacey et al., 2011; Weiss et al., 2011). The differential regulation of the inflammatory response induced by viable *C. pseudodiphtheriticum* seems to be related to its capacity to modulate TLR3-mediated inflammatory response in the respiratory tract. As others and we demonstrated previously, TLR3 has little effect on RSV clearance but it is necessary to regulate the respiratory immune environment. The absence of an efficient regulation of TLR3 activation significantly contributes to the pulmonary immunopathology associated to RSV infection (reviewed in 42). In fact, respiratory administration of the TLR3 agonist poly(I:C) has been used to mimic the pro-inflammatory and physiopathological consecuences of RSV infections in the lung (Kitazawa and Villena, 2014).

It has also been reported that the exacerbated proinflammatory cytokine/chemokine response is skewed toward a T helper type 2 (Th2) immune response (Cervantes-Ortiz et al., 2016), and that IFN-γ-producing CD4^+^ and CD8^+^ T cells contribute to protection during RSV infection (Sun and Lopez, 2016). In our experiments, treatment with viable *C. pseudodiphtheriticum* also increased IFN-γ producing CD3^+^CD4^+^ cells in the lungs of infant mice. The improved numbers of CD4^+^IFN-γ^+^ cells and IFN-γ levels in lung tissue would activate pulmonary macrophages and dendritic cells and induce the enhancement of Th1 cellular response, contributing to the protection induced by *C. pseudodiphtheriticum*.

In line with our previous studies evaluating the effect of beneficial microbes on the susceptibility to viral infections (Villena et al., 2012; Chiba et al., 2013; Tomosada et al., 2013), we found here that the respiratory commensal bacteria *C. pseudodiphtheriticum* improves resistance to RSV infection through the modulation of IFN-β, IFN-γ, and IL-10.

We also demonstrated here that viable *C. pseudodiphtheriticum* significanlty reduced *S. pneumoniae* cell counts in lungs and prevented its dissemination into the blood of infant mice after the primary infection with RSV. The effect of *C. pseudodiphtheriticum* in reducing lung pneumococcal cell counts was modest compared with our own previous studies. We had reported that the nasal administration of viable *Lactococcus lactis* NZ9000 to adult and infant mice reduced in more than two log folds *S. pneumoniae* cell counts in lungs (Medina et al., 2008). Moreover, *C. pseudodiphtheriticum* evaluated in an infant mice model of primary pneumococcal infection also reduced in more than two log folds *S. pneumoniae* cell counts in lungs when compared to untreated controls (data not shown). Despite the modest results obtained here by measuring the burden of the pathogen in the respiratory tract, *C. pseudodiphtheriticum* treatment was able to significantly reduce lung tissue damage and bacterial dissemination into the blood stream. These findings are of importance because experimental and clinical studies (Hament et al., 2005; Weinberger et al., 2013; Smith et al., 2014; Cebey-Lopez et al., 2016) showed that enhanced lung injuries and elevated levels of bacteremia are critical factors that determine the severity of infection and the rate of mortality.

It could be speculated that the beneficial modulation of the inflammatory response during RSV and the reduced lung injuries induced by *C. pseudodiphtheriticum* administration would be related to the improvement of secondary pneumococcal infection. The different respiratory immune environment (such as the levels of IFN-β, IFN-γ, and IL-10 in the lungs) on *C. pseudodiphtheriticum*-treated mice at the moment of pneumococcal infection would induce an improved immune response and protection. Of note, *C. pseudodiphtheriticum* was able to enhance CD4^+^IFN-γ^+^ cells in the respiratory tract. It has been reported that IFN-γ early during acute *S. pneumoniae* pneumonia induces transcription of target genes in the lungs, which are critical for host defense (Gomez et al., 2015). In addition, *C. pseudodiphtheriticu* stimulated the production of IFN-β in CD45^+^SiglecF^+^ alveolar macrophages after poly(I:C) administration. Some studies have showed that IFN-β is involved in the protection against lung tissue injury as well in the control of pneumococcal dissemination into the blood during secondary pneumococcal pneumonia. No significant differences in *S. pneumoniae* counts in the lungs of IFNAR1^−/−^ and IFNAR1^+/+^ mice were observed after pneumococcal challenge. However, pneumococci were observed earlier and at higher numbers in blood samples of IFNAR1^−/−^ mice compared to wild-type animals (LeMessurier et al., 2013). More detailed studies are necessary to fully understand the immune mechanisms involved in the protection against secondary pneumococcal pneumonia induced by *C. pseudodiphtheriticum* 090104.

Interestingly, non-viable bacteria did not have the same protective effect. Non-viable *C. pseudodiphtheriticum* was unable to induce the increase of the numbers of CD45^+^SiglecF^+^IFN-β^+^ and CD4^+^IL-10^+^ cells neither the levels of IFN-β and IL-10 in the respiratory tract. Only increments in CD4^+^IFN-γ^+^ cells and IFN-γ levels were detected but there were significantly lower when compared with those induced by viable bacteria. These results suggest that *C. pseudodiphtheriticum* 090104 colonization of the nasopharynx is indeed needed for reducing RSV infection and secondary bacterial infection. In line with this finding, it has been reported that the presence of viable *Corynebacterium* spp. in the upper respiratory tract is necessary to protect against pathogens since antibiotic perturbations leading to the reduction of beneficial commensal bacteria such as *Dolosigranulum* spp. or *Corynebacterium* spp. increase the risk of respiratory infections of healthy children (Pettigrew et al., 2012; Teo et al., 2015). Genome sequence analysis of the 090104 strain showed that the bacterium has gene clusters encoding fimbrial subunits and sortase A that are proteins involved in the attachment of fimbria to the cell surface (Karlyshev and Melnikov, 2013). To obtain mutants depleted from these genes and study whether these *C. pseudodiphtheriticum* strains lacking attachment proteins are able to beneficially modulate respiratory immunity is an interesting topic that we intend to evaluate in the immediate future.

In conclusion, we present evidence that nasal application of viable *C. pseudodiphtheriticum* could be thought as an alternative to boost antiviral defenses against RSV and secondary pneumococcal pneumonia, which should be further studied and validated in clinical trials. Due to the absence of a long-lasting immunity, re-infection with RSV throughout life is common. Thus, a possible perspective use could be a seasonal application of a nasal next-generation probiotic spray to boost respiratory innate immunity in immunocompetent subjects.

## Author contributions

SA, VM, HK, and JV designed the study. MV, HT, HK, and JV wrote the manuscript. PK, PC, MV, and CR did the laboratory work. PK, PC, and JV performed statistical analysis. HT, MV, and JV contributed to data analysis and interpretation. All authors read and approved the manuscript.

### Conflict of interest statement

The authors declare that the research was conducted in the absence of any commercial or financial relationships that could be construed as a potential conflict of interest.
